# Chronobiology of *Viscum album* L.: a time series of daily metabolomic fingerprints spanning 27 years 

**DOI:** 10.3389/fphys.2024.1396212

**Published:** 2024-05-27

**Authors:** Greta Guglielmetti, Stephan Baumgartner, Claudia Scherr, David Martin, Alexander L. Tournier

**Affiliations:** ^1^ Institute for Integrative Medicine, University of Witten/Herdecke, Herdecke, Germany; ^2^ Hiscia Research Institute, Society for Cancer Research, Arlesheim, Switzerland; ^3^ Institute of Complementary and Integrative Medicine, University of Bern, Bern, Switzerland

**Keywords:** chronobiology, mistletoe, metabolomic fingerprinting, pattern formation, image analysis, medicinal plants

## Abstract

**Introduction:** European mistletoe (*Viscum album* L.) has been gaining increasing interest in the field of oncology as a clinically relevant adjunctive treatment in many forms of cancer. In the field of phytopharmacology, harvesting time is pivotal. In the last century, a form of metabolomic fingerprinting based on pattern formation was proposed as a way to determine optimal harvesting times to ensure high quality of mistletoe as raw material for pharmaceutical use. In order to further evaluate the information obtained with this metabolomic fingerprinting method, we analysed a large time series of previously undigitised daily mistletoe chromatograms dating back to the 1950s.

**Methods:** These chromatograms were scanned and evaluated using computerized image analysis, resulting in 12 descriptors for each individual chromatogram. We performed a statistical analysis of the data obtained, investigating statistical distributions, cross-correlations and time self-correlations.

**Results:** The analysed dataset spanning about 27 years, contains 19,037 evaluable chromatograms in daily resolution. Based on the distribution and cross-correlation analyses, the 12 descriptors could be clustered into six independent groups describing different aspects of the chromatograms. One descriptor was found to mirror the annual rhythm being well correlated with temperature and a phase shift of 10 days. The time self-correlation analysis showed that most other descriptors had a characteristic self-correlation of ∼50 days, which points to further infradian rhythms (i.e., more than 24 h).

**Discussion:** To our knowledge, this dataset is the largest of its type. The combination of this form of metabolomic fingerprinting with the proposed computer analysis seems to be a promising tool to characterise biological variations of mistletoe. Additional research is underway to further analyse the different rhythms present in this dataset.

## 1 Introduction

Mistletoe is a plant of great interest in ethnopharmacology. In European traditional medicine, the use of the plant covers a wide range of indications, such as hypertension, atherosclerosis, internal bleeding, epilepsy, dizziness, anxiety, asthma, infertility and pain (Ogal, 2005; Gupta et al., 2012; Singh et al., 2016; Suveren et al., 2017; Szurpnicka et al., 2020). From the first decades of the last century onwards, the medical interest in this plant increased significantly due to a possible new application in cancer treatment (Steiner, 1961). In the 1920s, Steiner and Wegman, the founders of anthroposophic medicine (Steiner and Wegman, 1925), were the first to suggest the hypothesis of using mistletoe for cancer treatment. Since then a large number of clinical studies have been carried out with positive results for improving quality of life as well as for increasing survival (Büssing, 2000; Kienle and Kiene, 2003; Maldacker, 2006; Kienle and Kiene, 2007; Melzer et al., 2009; Ostermann et al., 2009; Kienle et al., 2011; Loef and Walach, 2020; Ostermann et al., 2020).

*Viscum album* L., commonly known as European mistletoe, is a perennial evergreen plant belonging to the Santalaceae family. It is a hemiparasite shrub, which depending on subspecies grows on different host trees. In particular, *V. album* ssp. *album* L. grows on broadleaf trees such as apple trees (*Malus domestica* Borkh.) among many others (Ramm et al., 2000; Zuber, 2004).

Pre-clinical studies on mistletoe extracts have shown the presence of chemical compounds which have been found to possess not only anti-tumour (both cytotoxic and anti-angiogenic) but also immunomodulatory activity: mainly lectins and viscotoxins but also alkaloids, triterpenes, oligo- and polysaccharides (Büssing, 2000; Kienle and Kiene, 2003; Maldacker, 2006).

The pharmacologically highly active mistletoe lectins and viscotoxins are considered the most important components of mistletoe preparations (Büssing, 2000) but their presence in the final product depends on many factors (choice of plant material, extraction procedure and pharmaceutical processes) (Urech and Baumgartner, 2015). In particular, it has been shown that the concentration of these two substances varies greatly throughout the year, with maximum concentrations of viscotoxins and lectins in June and December, respectively (Urech et al., 2009).

Chronobiological variations in plants are of special interest in phytomedicine. These should be taken into account to determine the most favourable harvesting time and ensure the final medicinal product has the optimum effect. Nevertheless, the growth/ripening processes of plants are also influenced by environmental factors (Upadhyay et al., 2022) which vary greatly depending on geographical position. For this reason, precise standardized recommendations do not exist. Harvesting time is therefore determined by farmers’ experience and/or by the identification of certain desirable molecules, as in the case of mistletoe (Büssing, 2000; Baumgartner et al., 2014).

However, in order to reach a deeper understanding of given plants’ chronobiology, we require daily tracking of the plant. Of particular interest is the metabolome, defined as the whole spectrum of chemical components (primary and secondary metabolites), whose fingerprint can be studied using metabolomic techniques. The combination of daily measurements and metabolomic fingerprinting enables a comprehensive study of also infradian rhythms, complementing known seasonal effects.

**FIGURE 1:**
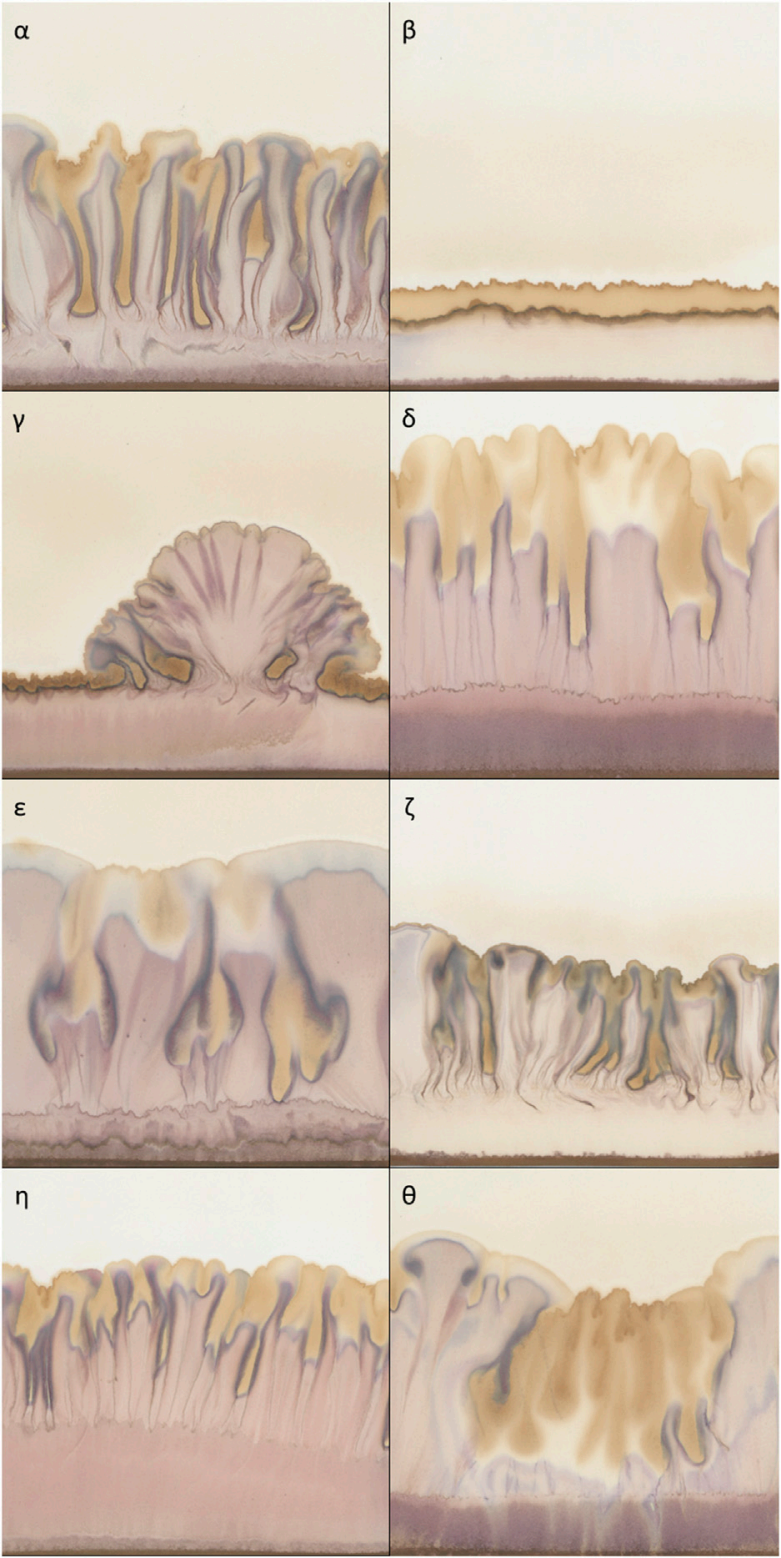
Typical examples of chromatograms of 50% extracts of *Viscum album* L. with AuCl_3_ as reagent, used as metabolomic fingerprints. **(α)** homogeneously risen chromatogram; **(β)** not risen chromatogram; **(γ)** heterogeneously risen chromatogram; δ) chromatogram with a wide purple band in the lower part; **(ε)** chromatogram with a typical distribution of yellow colour (top and central section); **(ζ)** chromatogram with a compressed aspect; **(η)** chromatogram with elongated aspect and yellow colour only at the top; **(θ)** chromatogram with a massive presence of yellow colour.

Anthroposophic medicine, as a whole systems medicine including phytomedicine, is very attentive to chronobiological phenomena. Indeed chronobiological aspects of mistletoe have been studied for decades (Fyfe, 1969; Scheer et al., 1992; Flückiger and Baumgartner, 2002; Dorka et al., 2007; Urech et al., 2009; Derbidge et al., 2013; Derbidge et al., 2016). One of the pioneers of such studies was Agnes Fyfe, a researcher at Hiscia Research Institute (Society for Cancer Research, Arlesheim, Switzerland). Already in the 1950s, with the aim of identifying optimal harvesting times, she started to build up a dataset of chromatograms using a form of metabolomic fingerprinting based on pattern formation (hereafter referred to as metabolomic fingerprinting) peculiar in terms of ease, rapidity and affordability. She conducted daily chromatograms of *V. album* ssp. *album* L. for nearly 30 years (from November 1958 to October 1985) creating a unique heritage. This set is called “Fyfe dataset” and it consists of two subsets: “Gold Fyfe dataset” and “Silver Fyfe dataset” as the researcher performed the experiments with two different reagents, AuCl_3_ and AgNO_3_, respectively (see *Methods*). In this publication we will focus on the “Gold Fyfe dataset”. Mistletoe samples were collected daily, and their juice was extracted and promptly analysed. In this way, 27,979 chromatograms were gathered showing recurring variations of different patterns (Figure 1). Fyfe interpreted the observed fluctuations in the patterns in terms of the “quality” of the mistletoe extract, based on the absence/presence of certain features through comparative visual evaluation (Fyfe, 1975).

To the best of our knowledge, no other plant has ever been monitored through daily experiments over such a long period, so the potential amount of information recorded in this dataset is remarkable. This fact piqued our interest and led to this investigation. Our study aim is to present this dataset along with a preliminary analysis using computerized image analysis to identify possible chronobiological rhythms.

## 2 Materials and methods

### 2.1 Metabolomic fingerprinting based on pattern formation

Fingerprint metabolomic analysis was performed making use of capillary suction of liquids on chromatography paper. In the standard setup, an upright cylinder of chromatography paper is placed into a Kaelin dish (analogous to a Petri dish, but with a circular groove). In the first phase of the process, 1 mL of the aqueous sample to be investigated (in our case mistletoe extract) is added to the dish and subsequently rises within the chromatography paper until all liquid is soaked up. Afterwards, the paper is allowed to dry. In the second phase, 2 mL metal salt solution (here 1% AuCl_3_) is added to the Kaelin dish and the liquid again rises within the chromatography paper until all liquid is soaked up. Subsequently, the paper dries again. This two-step process allows structures and shapes to emerge through the differential migration of chemical components on the paper creating patterns of different possible shapes and colours [for further specifications and a fully detailed description of the method refer to (Skjerbaek et al., 2004)].

These different patterns are interpreted to mirror the level of resilience of the sample (Athmann et al., 2021). This method as well as others [copper chloride crystallization (Busscher et al., 2010) and circular chromatography (Pfeiffer, 1984; Kokornaczyk et al., 2016)] can be classified as metabolomic fingerprinting based on pattern formation, as introduced by (Doesburg et al., 2019). When this method was invented by Lily Kolisko in the 1920s (Kolisko, 1953; Kolisko and Kolisko, 1978; Steffen, 1983), it was named capillary dynamolysis. Since then, many adaptations have been developed, mainly variations of the metal reagents used. Subsequently, a variation which is principally characterised by the use of two reagents (AgNO_3_ and FeSO_4_) which leads to completely different pictures to those of the dataset presented, has been standardised (Skjerbaek et al., 2004; Zalecka et al., 2010). Variations of the pattern formation based methods were also developed for and used in medical diagnosis (Kokornaczyk et al., 2021).

### 2.2 Experimental procedure

Samples of European mistletoe (*V. album* ssp. *album* L. growing on *M. domestica* trees in natural conditions in Arlesheim, Switzerland) were harvested on a daily basis between 1st November 1958 to 14th October 1985. *Viscum album* leaves and stems of the most recent generation in the ratio of 2:1 were used. In some cases, exceptions were made, as the notes provided at the top of the chromatograms hint at the use of other developmental stages (597 chromatograms, 3.12% of the selected extract concentration subset, see below). These are defined as young/old leaves or leaves from the current/past year. The harvesting time was always 8 a.m. apart from few exceptions. In 887 chromatograms the harvesting time is other than 8 a.m. and 878 chromatograms have no time specification (4.64% and 4.59% respectively of the selected extract concentration subset, see below). After wiping the sample from any excess water or dirt if necessary, it was cut into small pieces and ground to a paste using a mortar and pestle. Distilled water was added according to the desired final concentration (50% or 10%). The extract was obtained by squeezing the paste by hand through a wet linen cloth. Straight after, the extract was set to rise on the chromatography paper. As far as is possible to reconstruct from the available records, the vast majority of the chromatograms were made by Agnes Fyfe. However, in 1980 Heidi Flückiger joined the project and we know from an interview (Inhetveen et al., 2021) that she was trained by Fyfe for over 2 years (the actual number of experiments she performed is unknown).

All chromatograms were obtained using the following procedure (Fyfe, 1975). The laboratory was equipped to maintain about 70% humidity, was regulated at 20°C and had darkened windows. Chromatography paper (Whatman^®^ Nr.1, 14 cm × 17 cm, Cytiva, Marlborough, United States) was wrapped with a rolling machine specifically designed at the Society for Cancer Research for this project to ensure consistent folding conditions over time. Chromatograms were stored in booklets of firm paper in the dark. The following information was always recorded on each chromatography paper: sample name and concentration, harvest date and a numerical identification code. Every sample was often analysed using two extract concentrations (50% and 10%). Within the selected extract concentration subset (50%, see below), for 85% of the experimental days, multiple chromatograms were obtained (with up to eight replicates per day).

### 2.3 Scanning of chromatograms

All 27,979 chromatograms were scanned between 23.06.2020 and 02.04.2021 in reflective mode using an Epson Perfection V600 scanner (Epson, Kloten, Switzerland). Since the chromatograms are colour pictures, colours were calibrated using: IT 8.7/2 RF target (Wolf Faust, Frankfurt, Germany) and SilverFast Ai Studio 8.8 (LaserSoft Imaging AG, Kiel, Germany) as calibration program. Images were saved in TIFF format, 300 DPI, RGB, 16 cm × 17 cm.

### 2.4 Inclusion and exclusion criteria

**FIGURE 2:**
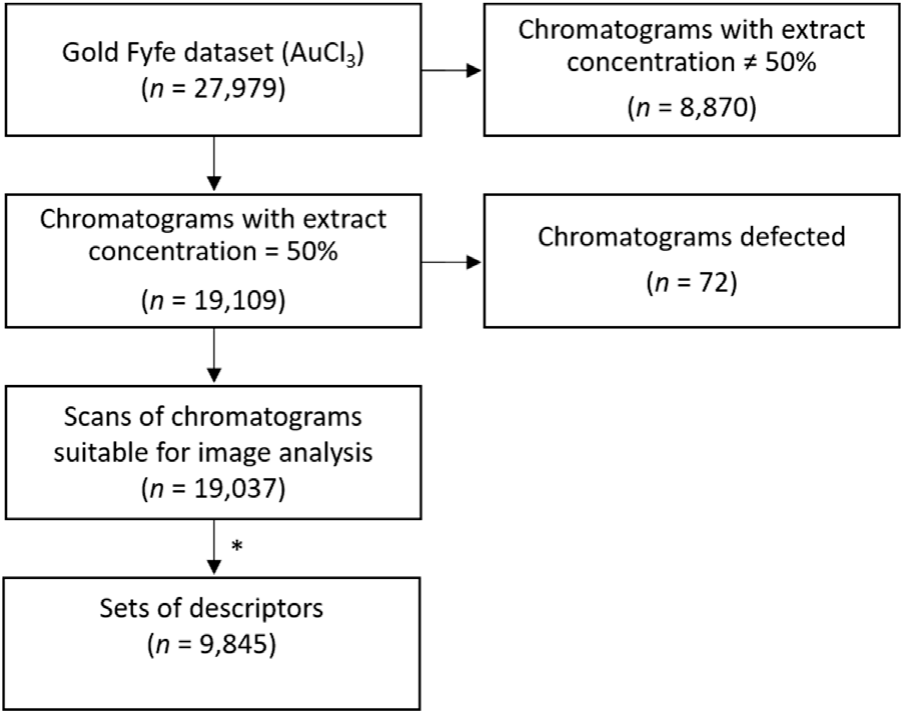
Flow diagram of data pipeline and inclusion/exclusion criteria of the chromatograms for the analysis. * Among the scans of chromatograms suitable for image analysis, if two or more daily replicates were available, corresponding data of the twelve descriptors were averaged. We thus obtained a single set of the twelve descriptors per day, ending up with 9,845 sets of daily descriptors.

Chromatograms made with 50% mistletoe extract concentration (*n* = 19,109) were selected for this study as they showed clearer visual characteristics suitable for image analysis than those made with 10% mistletoe extract (*n* = 8 870). Among these, an exclusion criterion was based on the ability of ImageJ analysis to analyse chromatograms on old/damaged paper. Those images for which the analysis did not manage to find their outline were discarded (40 chromatograms), as well as those in which an outline was found but which did not match at all the actual one upon visual inspection (32 chromatograms). The final dataset consisted of 19,037 chromatograms. When multiple chromatogram replicates per day were available, data obtained in the image analysis were averaged to daily means (see below) ending up with 9,845 days with available data for analysis (Figure 2). Over the about 27 years of daily data gathering, only 226 days were missing (2.35%).

### 2.5 Image analysis

Image analysis was entirely performed in batch using Fiji ImageJ 2.1.0/1.53c; Java 1.8.0_172 (Schindelin et al., 2012).

**FIGURE 3:**
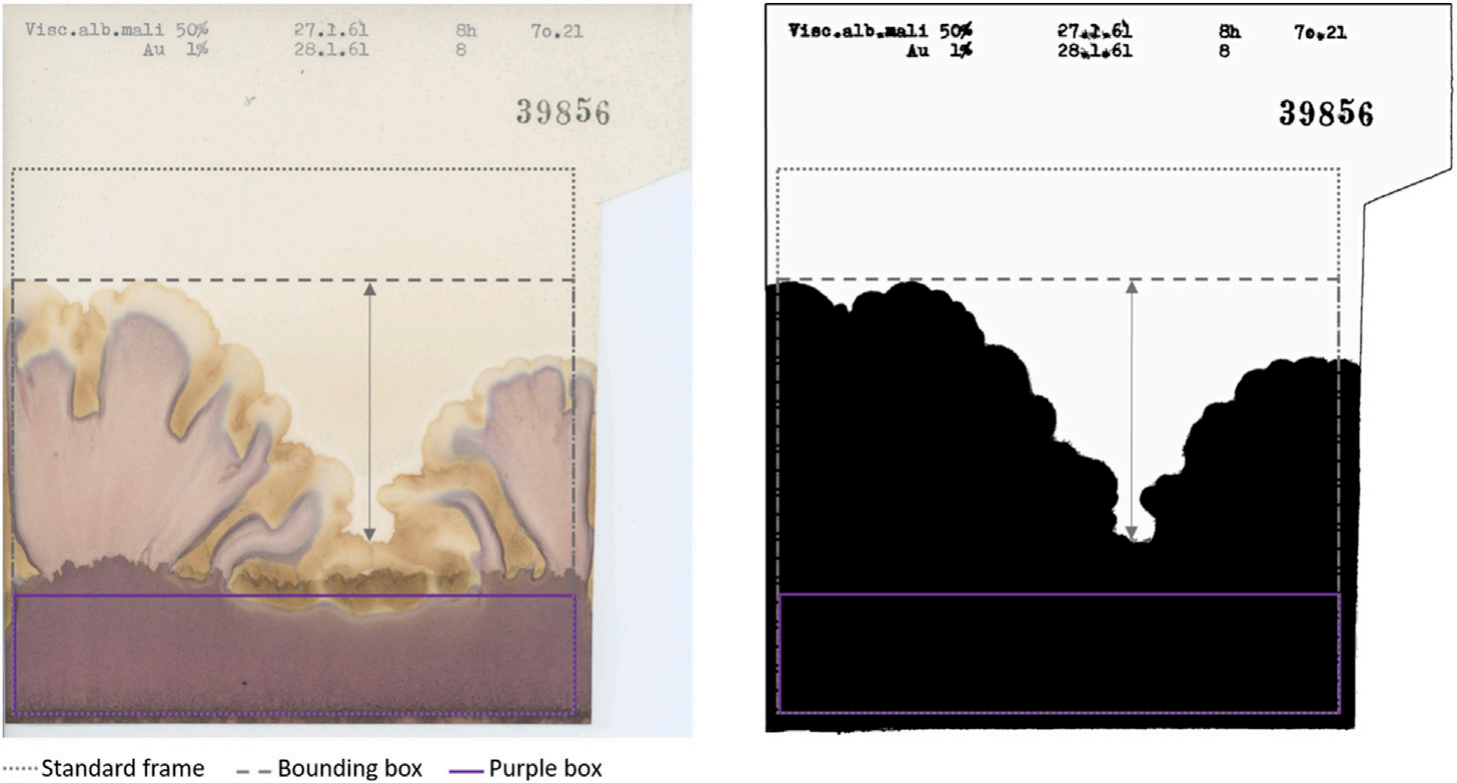
Original (left) and processed outline (right) of a representative chromatogram included in the “Standard frame” [dotted frame at position (36, 428), size (1,533, 1,477)] and in the “Bounding box” (dashed frame different for each chromatogram). The arrow represents “Prominence” defined as the difference between the highest and lowest peak. “Purple box” [purple frame at position (0, 1,188), size (1,533, 289)] is the region of interest for purple colour analysis. The area above the “Purple box” contained within the “Bounding box” is the region of interest for yellow colour analysis [position (0,0), size (1,533, 1,170)].

As a first step, a fixed region of interest called “Standard frame” was defined (indicated by the dotted frame in Figure 3) as wide as possible but which excluded critical areas, namely, the edges, which represented a problem for image analysis. As can be seen in Figure 3, the upper edge of the picture contains written information about the experiment. The left, right and bottom edges of the chromatography paper would often be torn/broken due to age (about 40 to 60 years old).

**FIGURE 4:**
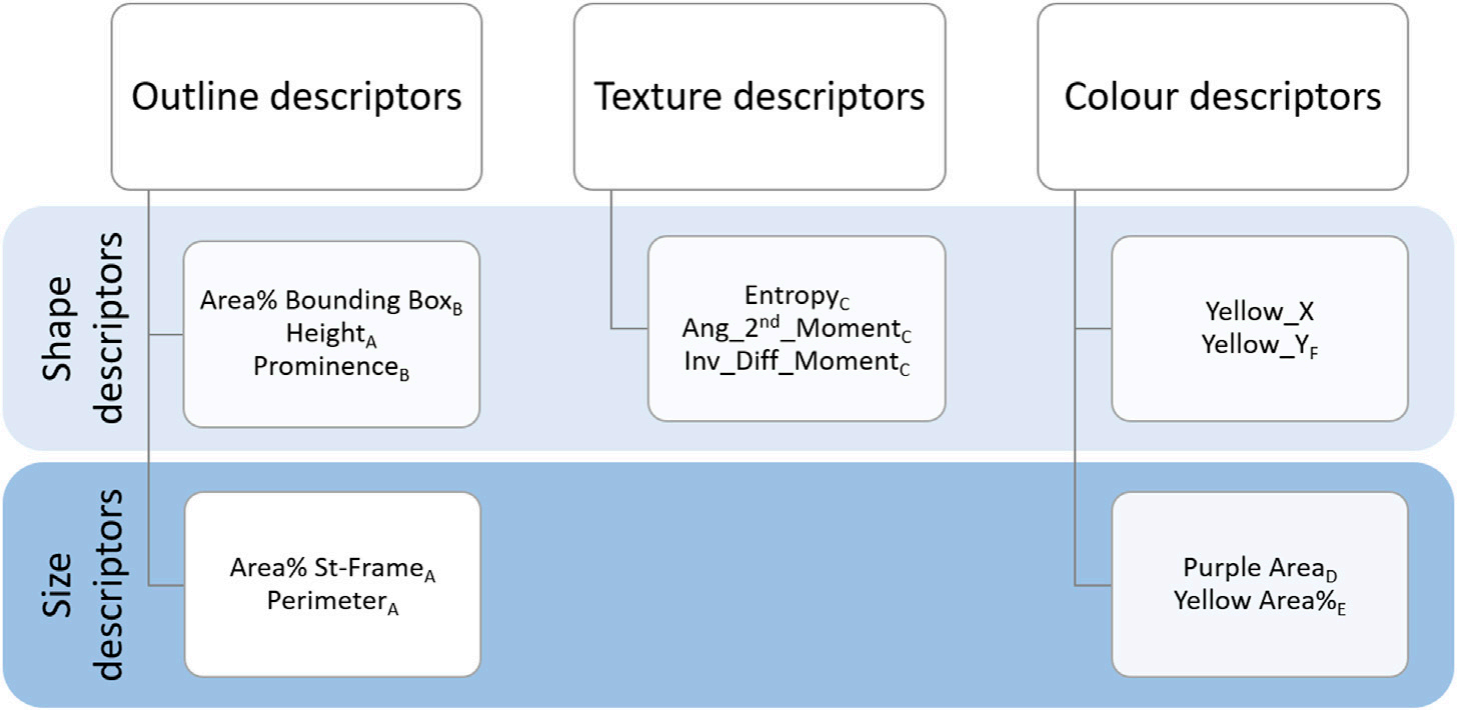
Summary of the descriptors. Outline, Texture and Colour descriptors are listed by their definition to describe the shape or size of the chromatograms. Subscripts from A to F represent the distribution groups to which descriptors belong (see Figure 5).

We focussed on three different image analysis strategies (see Figure 4). The first one characterises the outline of the chromatograms, the second one performs a texture analysis and the last one describes colours. They provide useful tools to describe different aspects of the shapes and colours present in the chromatograms. All three analyses were applied to the data contained in the “Standard frame” (frame identical for all chromatograms).

#### 2.5.1 Outline descriptors

As shown in Figure 4, Outline descriptors can be divided into two groups according to the aspect they characterise. As a basic approach, we calculated features able to describe the size of the outline, namely, the area and the perimeter within the “Standard frame” (dotted frame in Figure 3).

The main ImageJ tool used in this step was Find Edges. This, combined with contrast adjustments before (pixel saturation = 10) and after (pixel saturation = 6) the application of a filter (Gaussian blur, sigma = 2) and subsequently thresholding (Shanbhag) the blue channel of the RGB image, made it possible to distinguish the outline of the chromatogram from the background. Any small particles which interfered with outline identification were removed using a combination of Analyse Particle and Erosion tools switching the selection from the background (particle size 1500-Infinity) to the foreground (particle size 100000-Infinity). As a result, a mask (i.e., binary image shown in Figure 3 right) of the outline was obtained. We then could calculate the first two descriptors:

• **Area% St-Frame**: percentage of the area occupied by the black outline within the “Standard frame.”
• **Perimeter**: perimeter of the black outline within the “Standard frame.”

Secondly, in order to provide a more thorough description of the outline of the chromatograms, we calculated parameters characterising the shapes present in the pictures. These varied depending on whether the rise was homogeneous or heterogeneous (Figure 1γ shows a heterogeneous rise compared to the other chromatograms shown in Figure 1). The following Outline descriptors were no longer calculated in relation to the “Standard frame” but to the “Bounding box” of the mask, which is the smallest rectangular frame in which an object can be contained (indicated in Figure 3 with the dashed line). Using the selection plugin To Bounding Box, we obtained a frame of a different size for each chromatogram. Within this new frame, the percentage of the area occupied by the outline was again calculated (“Area% Bounding Box”), as well as the height of it (“Height”) which corresponds also to the height of the “Bounding box” (in Figure 3 visible as the vertical component of the dashed frame). Moreover, also the “Prominence,” defined as the height difference between the highest and the lowest peak was calculated. It is represented by the arrow in Figure 3. The calculation of it was made by focusing the selection on the background of the “Bounding box” and calculating its height.

To summarise, the outline shape has been characterised by the following descriptors which relate to the black outline within the dashed frame (“Bounding box”) in Figure 3:

• **Area% Bounding Box**: percentage of area occupied by the black outline within the “Bounding box.”
• **Height**: height of the “Bounding box” which corresponds to the maximum height of the outline.
• **Prominence**: height difference between the highest and the lowest peak of the outline.

#### 2.5.2 Texture descriptors

Texture descriptors characterise the structures present within the chromatograms (e.g., the difference between Figure 1η and ζ) (O’Byrne et al., 2012). When we visually examined the dataset we noticed that in some cases, regardless of the height reached, the chromatograms developed more compressed structures with a very pale pink but dark yellow (Figure 1ζ). In other cases, the structures were more elongated and with brighter colours (Figure 1η). In order to capture these characteristics, we used the Texture Analyser plugin (Cabrera, 2006). Images were converted to 8-bit, Find Edges tool was applied and contrast was adjusted (pixel saturation = 1). This allowed us to highlight the contours of the structures. Lastly, the Texture Analyser was run. The following parameters were computed as proposed by Haralick (Haralick et al., 1973):

• **Entropy**: measurement of the randomness of the intensity distribution of the image.
• **Ang_2**nd**_Moment**: measurement of the uniformity of the distribution of the grey scale of the image.
• **Inv_Diff_Moment**: measurement of the local homogeneity of the image.

#### 2.5.3 Colour descriptors

One of the most striking features of these chromatograms is the variety of colours produced, especially purples and yellows, and their arrangement in the chromatography paper. The plugin called Trainable Weka Segmentation (Arganda-Carreras et al., 2017), combines several machine-learning algorithms applied to specific image features. Using this segmentation tool, we defined the classes for purple and yellow colours. The characterisation of the classes was done manually by an operator (GG) who trained the algorithm. Running in batch the segmentation according to the defined classes, we obtained as outcome of this analysis a probability map for each class. The purple segmentation map was used to calculate the first colour-related descriptor: “Purple Area.” Since the characterisation of the chromatograms can be based just on the presence/absence of purples in the bottom region, as visible in Figure 1δ vs ζ, it was sufficient to determine the size of the area covered by it. We selected a rectangular region of interest at the bottom of the chromatograms where a purple band possibly appears (purple frame called “Purple Box” in Figure 3) and the area was calculated. After the conversion to 8-bit, a threshold (Default) had been applied and the area was calculated.

Regarding yellow-related descriptors, a rectangular region of interest which covered the central and upper part of the pictures (in Figure 3 indicated as the area above “Purple Box”) was selected on the yellow segmentation map, and the area calculated (“Yellow Area%”). The area covered by yellow can be bigger or smaller (Figure 1θ and ζ respectively) but it can also have different locations on the chromatography paper (relegated on the top as Figure 1β and η or distributed also in the central section of Figure 1α and ε) creating different patterns. Therefore, we calculated “Yellow_Y” and “Yellow_X” which are respectively the vertical and horizontal components of the centre of mass of the yellow area. “Yellow_X” was used as a negative control as it should be randomly distributed around the centre of the paper. This is because the liquids (sample and reagent), whose interaction generates the colours, are set to rise along the whole sheet of chromatography paper. Therefore, the X-component of the centre of mass of the colours should be randomly distributed around the centre of the paper.

To summarise, the following colour descriptors were calculated:

• **Purple Area**: area in pixels covered by purple colour in the bottom section of the chromatograms.
• **Yellow Area%**: percentage of area covered by yellow within the region of interest above the “Purple Box.”
• **Yellow_Y**: vertical component of the centre of mass of the yellow area.
• **Yellow_X**: horizontal component of the centre of mass of the yellow area.

### 2.6 Weather data

Meteorological data of the same time span as our dataset (1958–1985), measured at the meteorological station of Basel-Binningen, were obtained from MeteoSwiss (Switzerland). With 8 km distance, Basel-Binningen is the nearest official weather station to Arlesheim where mistletoe was collected. Among the 105 meteorological parameters available, we discarded those for which data availability was less than 80% of our time period, resulting in 38 parameters that were used as independent variables to investigate potential correlations with our descriptors (see Supplementary Table S1).

### 2.7 Data analysis

All the data collected were processed and statistically analysed using Python (Version 3.10, https://www.python.org/). The data was analysed as a time series, with one entry per day. In cases where two or more chromatogram replicates were available, corresponding data were averaged in order to obtain a single set of the twelve descriptors per day. From 19,037 chromatograms we thus ended up with 9,845 sets of daily descriptors. The frequency distribution, cross-correlation between descriptors, self-correlations and correlation with weather variables were calculated.

## 3 Results

### 3.1 Data distributions

**FIGURE 5:**
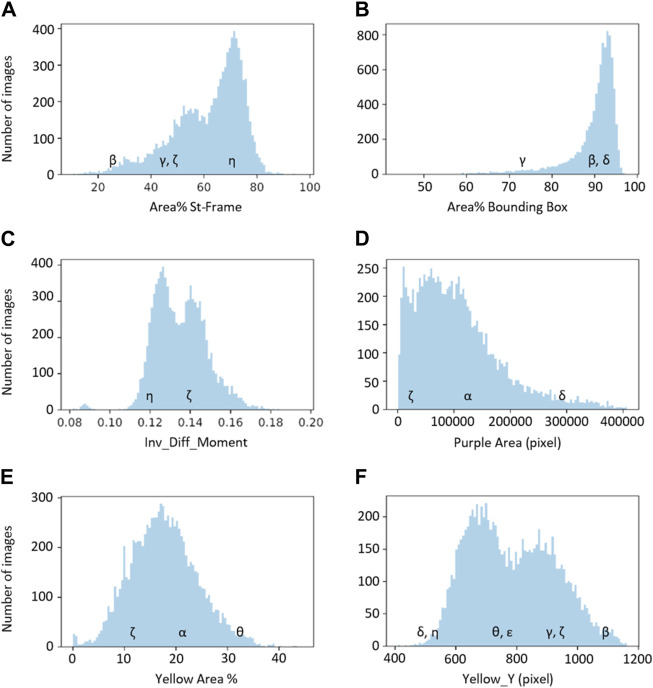
The six basic groups of data distribution **(A–F)** of the chromatogram descriptors. Greek letters referring to the chromatograms of Figure 1 are superimposed on the histograms to exemplify the meaning of the chromatogram descriptors.

Descriptors could be clustered into six groups according to similarities in their distributions (see histograms in Figure 5; also indicated by the letters as subscripts in Figure 4).

The first group, group A, includes three descriptors with similar histograms: “Area% St-Frame,” “Height” and “Perimeter” (represented by “Area% St-Frame” in Figure 5A). The remaining Outline descriptors, namely, “Area% Bounding Box” and “Prominence,” belong to group B, which is represented by the histogram for “Area% Bounding Box” in Figure 5B. All three Texture descriptors (“Entropy,” “Ang_2nd_Moment” and “Inv_Diff_Moment”) show a similar distribution; “Inv_Diff_Moment” in Figure 5C is representative of this group. The distributions of the Colour descriptors are more diverse, so each descriptor forms a group by itself: group D for “Purple Area,” group E for “Yellow Area%” and lastly, group F for “Yellow_Y” (Figures 5D–F).

The distributions of groups B, D and E are characterised by the presence of one major population, although with different widths and specific shapes. On the other hand groups A, C and F show the presence of two subpopulations (with a smaller third population for distribution A).

Examples of characteristic chromatograms corresponding to different values of the descriptors are referenced in the histograms of Figure 5 as Greek letters, with reference to Figure 1.

“Yellow_X,” being the negative control, has not been included in the grouping.

### 3.2 Cross-correlations between chromatogram descriptors

**FIGURE 6:**
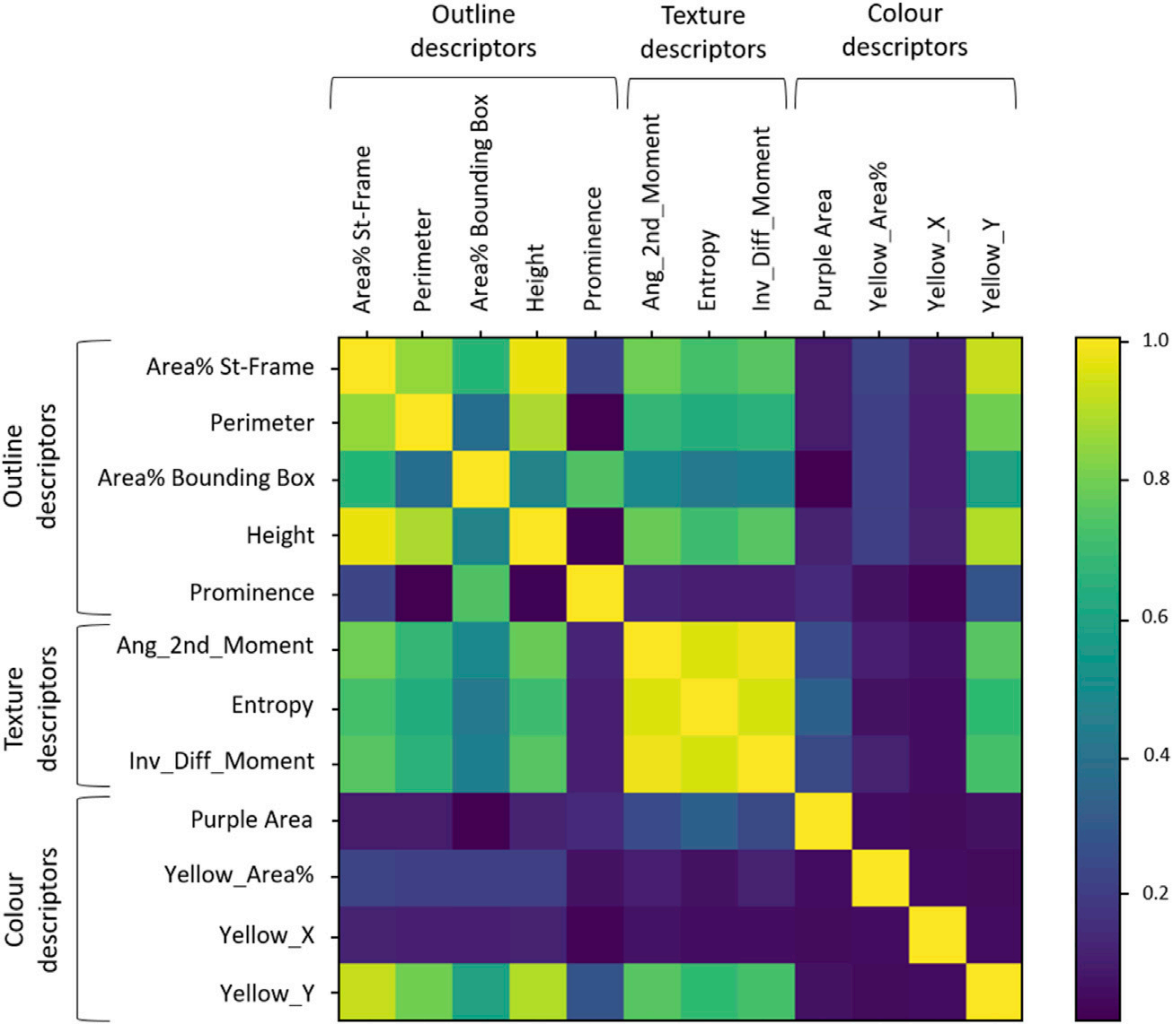
Cross-correlation matrix between descriptors. The coloured bar on the right gives grades of colours as reference for highest (1 = yellow) to no correlation (0 = deep blue).

The cross-correlation between descriptors is shown in Figure 6. Descriptors belonging to the Texture category were correlated with each other (r > 0.9). The descriptors belonging to the Outline category were also correlated to each other but to a lesser extent, while the Colour descriptors were more independent from one another.

Looking more closely at the Outline descriptors, two groups of correlated descriptors were visible: group A (“Area% St-Frame,” “Perimeter” and “Height”) and B (“Area% Bounding Box” and “Prominence”) of Figure 5 were in line with the distribution analysis observations. The groupings based on the distribution analysis were also confirmed for the Texture descriptors and Colour descriptors: high correlation between the Texture descriptors and independence of the three Colour descriptors. A new aspect that could be observed in Figure 6 was the correlation of “Yellow_Y” with Outline descriptors and Texture descriptors. We also observed the expected independence of the negative control (“Yellow_X”).

### 3.3 Self-correlation of descriptors

**FIGURE 7:**
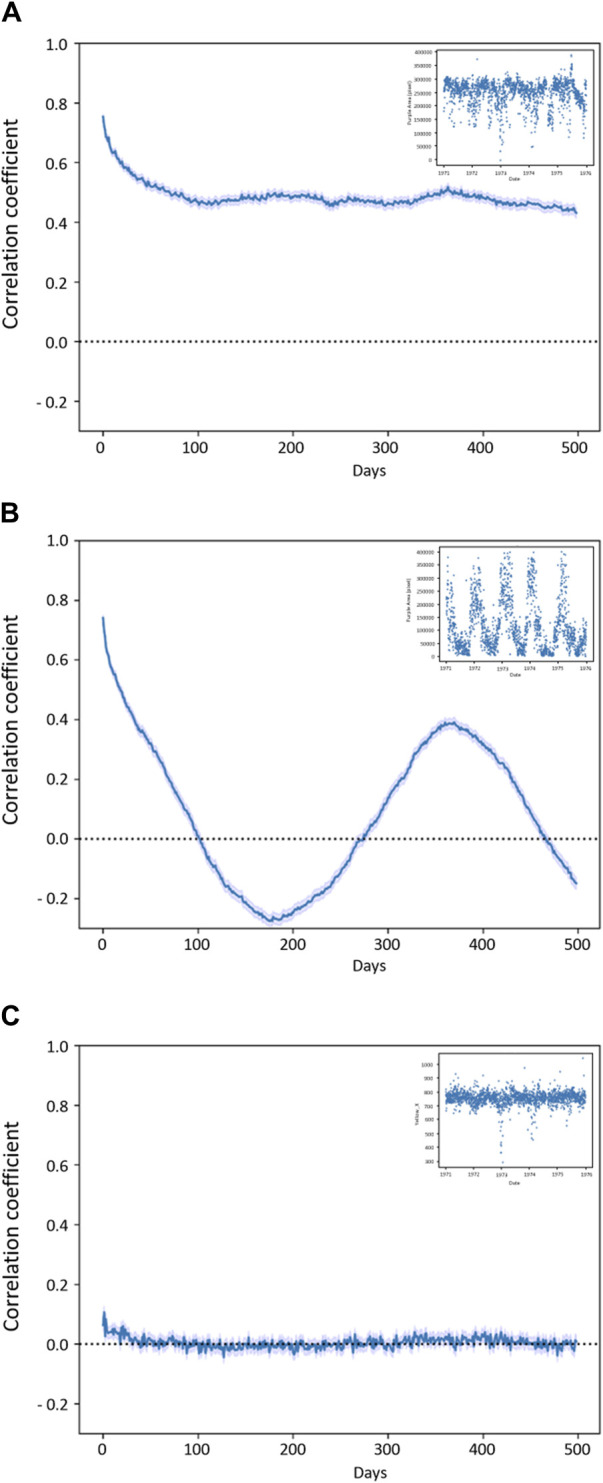
Self-correlation plots **(A)** plot “Area% St-Frame” (representative for “Height”, “Entropy”, “Ang_2nd_Moment”, “Inv_Diff_Moment” and “Yellow_Y”) shows a high correlation within the first 50 days; **(B)** “Purple Area” self-correlation with yearly trend; **(C)** “Yellow_X” with no self-correlation as expected from the control. In the small box, a short frame of 5 years (1971–1976) is provided for each graph to show the behaviour of the descriptors in the course of time, in the case of “Purple Area” the yearly rhythm is visible.

The self-correlations of most descriptors started at higher values and tended to drop over the first 50 days, and then slowly decreased until reaching 0.0 over the years (see Figure 7A, “Area% St-Frame” as a typical example). “Area% St-Frame,” “Height,” “Entropy,” “Ang_2nd_Moment,” “Inv_Diff_Moment” and “Yellow_Y” showed the highest self-correlations (initial r = 0.8) with a characteristic timescale of ∼50 days. “Perimeter” and “Yellow Area%” showed less initial self-correlation (initial r = 0.6) with the same ∼50-day timescale. Finally, “Area% Bounding Box” and “Prominence” showed lower initial self-correlations (r = 0.4 and 0.2 respectively), but with the same characteristic timescale of ∼50 days. A different pattern was present in the case of “Purple Area” which showed a clear sinusoidal pattern over time (periodicity 1 year) with a high initial self-correlation of 0.8 (Figure 7B). The control, “Yellow_X” showed no self-correlation as expected (Figure 7C).

### 3.4 Annual rhythm

The yearly periodicity observed for “Purple Area” led us to investigate potential correlations with weather parameters as they would be expected to have annual rhythms. Indeed, high correlations were found (data not shown) between certain weather parameters and this descriptor indicating a correlation between the presence of the purple-coloured band at the bottom of the chromatograms and temperature-related parameters. In particular, a significant correlation (*p* < 0.001 between 0.38 and 0.41 was found for temperature and vapour pressure (ths200dx, ths200dn, ths200d0, tre200dx, tre200dn, tre200d0, pva200d0 in Supplementary Table S1).

**FIGURE 8:**
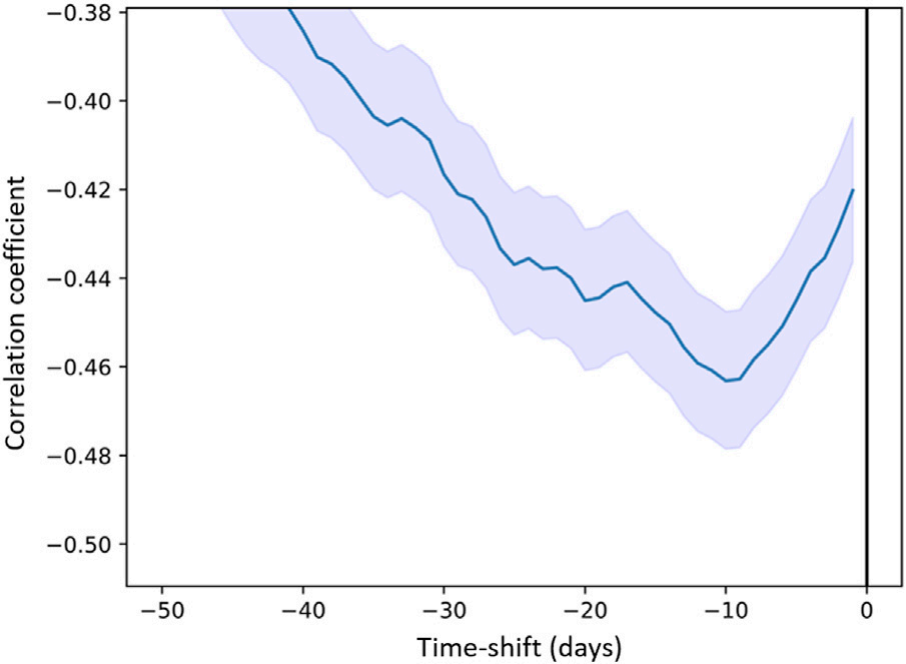
Correlation coefficient between “Purple Area” (descriptor) and air temperature 2 m above ground as daily mean “tre200d0” (predictor) as a function of time (days) delay.

This correlation means that the purple area in the chromatograms increased with lower temperatures (i.e., wintertime). A detailed analysis revealed that there is a phase shift of 10 days, i.e., temperature changes occurred on average 10 days before the corresponding changes in the metabolomic fingerprints (Figure 8). Air temperature 2 m above ground as daily mean °C (tre200d0, Supplementary Table S1) was chosen as a representative temperature parameter in Figure 8.

## 4 Discussion

To our knowledge, we here present part of the largest metabolomic fingerprint dataset available in mistletoe chronobiology. The “Gold Fyfe dataset”— with a total 27,979 chromatograms daily made over 27 years—provides a solid basis for further analysis. The combination of Outline, Texture and Colour descriptors allows to describe different aspects of the chromatograms obtained. These descriptors clustered into six independent groups, based on the histograms and cross-correlation analysis. Further confirmation of the validity of our descriptors was shown through the time self-correlation analysis which revealed that the signals were not random, showing a self-correlation over about 50 days, pointing towards infradian rhythms. Noteworthy is also “Purple Area” which showed a clear yearly rhythm. The correlation of “Purple Area” and temperature confirmed the emergence of the most easily detectable periodicity in the plant realm, namely, the seasonal rhythm. Furthermore, the 10-day phase shift observed between the temperature change and the pattern variation in the chromatograms supports the biological plausibility of the correlation as it represents a reasonable interval for a plant to respond to temperature variations (Nievola et al., 2017).

Literature about mistletoe chronobiology covers different aspects of the plant. The first investigations aimed to characterise the developmental stages of *V. album* using different forms of metabolomic fingerprinting (capillary dynamolysis and copper chloride crystallization) (Fyfe, 1969; Koopmans, 1972). Subsequently, the seasonal variation of specific chemical compounds (e.g., mistletoe lectins and viscotoxins) and their importance in cancer therapy was discovered (Scheer et al., 1992; Büssing, 2000; Urech et al., 2009). Other studies covered botanical and morphological aspects, such as those on nutational movements of the mistletoe bush as a whole (Dorka et al., 2007) as well as circadian and infradian rhythms in the shape of mistletoe berries (Flückiger and Baumgartner, 2002; Derbidge et al., 2013; Derbidge et al., 2016). Considering the discovery of these rhythmicities which affect different aspects of the plant and the medical interest mistletoe has been gaining as cancer treatment, deeper investigations should be performed to investigate if harvesting times can be honed. The presence of specific anti-cancer substances provides an orientation for harvesting time on a seasonal time scale. In fact, several anthroposophic mistletoe preparations consist of a mixture of summer and winter mistletoe in order to mirror in the final product the peaks in lectins and viscotoxins content (Baumgartner, 2016). Nevertheless, continuous and extensive observations of the whole metabolome should be conducted to clarify whether the metabolome is subject to variations similar to those we observed. For future investigations, the same could be tested on other pharmacologically relevant plants. In particular, a comparative study on other mistletoes would be interesting to check if common aspects may emerge. Of particular pharmacological interest might be the Loranthaceae family, which includes several species of mistletoe used in traditional medicine (Moghadamtousi et al., 2013; Omeje et al., 2014; Ameer et al., 2015; Sharquie et al., 2016; Ambrosio et al., 2020).

The “Gold Fyfe dataset” provides a precious and *sui generis* instrument not only to contribute to the question on possible infradian rhythms of *V. album* metabolome but also for chronobiology in general. In particular, the uniqueness of the dataset is visible in two aspects.

The first aspect concerns the basic features of the dataset in terms of resolution and duration (daily experiments over about 27 years). There are some datasets of experiments or observations comparable in terms of length which were conducted in the agricultural field, but in these cases, measurements are usually performed yearly or are harvest-dependent (Mader et al., 2002; Christensen et al., 2022; Krause et al., 2022), while the “Gold Fyfe dataset” has a daily resolution. Another huge database running from the 1960s which collects measurements of the concentrations of chlorophyll-α at Windermere Lake in the UK is comparable in terms of duration, but also in this case, the resolution is lower (fortnightly) (Hemming et al., 2018).

The second noteworthy aspect is that thanks to the daily resolution, the dataset specifically enables the study of infradian rhythms. There is extensive literature about rhythmic oscillations of roughly 24 h or less (i.e., circadian and ultradian rhythms, respectively) in plants (Mancuso and Shabala, 2006; McClung, 2006; Venkat and Muneer, 2022). Fewer studies focus on plant infradian rhythms and all of the studies we know are characterized by a shorter duration and/or lower frequency of experiments (Lindholm, 1990; Gerdol, 1996; Asada et al., 2003; Buda et al., 2003; Aono and Kazui, 2008; Yazaki and Yabe, 2012; Barlow et al., 2013; Mironov and Kondratev, 2017; Mironov et al., 2020).

Thus, to the best of our knowledge, the “Gold Fyfe dataset” is unique in chronobiology in terms of length and resolution.

We were able to detect seasonal rhythms in the “Gold Fyfe dataset”, however, it is not the only signal that seems to be present in the data. In fact, apart from the negative control, all the other descriptors show some auto-correlation, corresponding to non-random signals, indicating that there are possibly other infradian rhythms present within this dataset. It was beyond the scope of the present investigation but will be topic of future data analysis to test the correlation of these signals with a variety of external factors other than weather and climate and to apply further signal analysis techniques. Moon rhythms but also geomagnetic events have been shown to be relevant triggers in chronobiology (Barlow et al., 2013; Mironov and Kondratev, 2017; Mironov et al., 2020; Sivasankar and Thimmaiah, 2021).

Our results highlight many intriguing aspects not only about the samples but also about the method itself. In this regard, there appears to be great potential for this form of metabolomic fingerprinting as it proved sensitive, easy and fast. Moreover, the novel computer analysis we propose in this paper constitutes a significant improvement of the method so that it can play a role in modern chronobiological studies. Computer analysis of chromatograms obtained with a variation of this method (using two metal reagents instead of one) was previously performed (Unluturk et al., 2011; Larsen et al., 2013; Unluturk et al., 2021). However, the completely different appearance of the chromatograms makes comparability impossible and therefore also the application of the same computer analysis is not possible.

We are aware there are some limitations of this dataset. One of the main problems is the lack of constant and precise information regarding each chromatogram in view of current reporting standards. For example, climate information known to influence capillary dynamics (temperature and humidity) was declared to be maintained stable (Fyfe, 1975) but it was not reported on each chromatogram, making double-check not possible. Another important methodological limitation is the manual juice extraction which does not ensure defined consistency in the extraction process. That being said, we know that just one person carried out the chromatograms for most of the time, thus ensuring consistency; and only in the last few years, and after thorough training, did another researcher take over. Nevertheless, we did find a strong correlation with seasonal rhythms pointing to the fact that even if such inaccuracies and operator variations existed, they did not interfere with our ability to detect seasonal and other rhythms present in the pictures. Another aspect which should be considered concerning the methodological side of this kind of metabolomic fingerprinting is to understand if and how chemical/physical parameters (e.g., viscosity) of the sample can influence the development of the pattern, as this may be reflected in the parameters we calculated and new correlations may emerge. Lastly, more sophisticated image analysis would be beneficial to further characterise the different populations of chromatograms already discovered or to discover new ones. Improvements in texture analysis, which could provide information about colour arrangements, or a more complex analysis taking account of several features simultaneously, for example, as machine learning techniques, would be desirable.

## 5 Conclusion

In this article, we present and characterise the largest metabolomic fingerprint time series with a daily resolution of a plant (*V. album*). We also propose a novel computer-based analysis for the characterization of the chromatograms obtained with this method. The combination of the method and the computer analysis has proved successful in detecting chronobiological variations in *V. album*: not only the expected seasonal cycle but also other signals seem to be present in the dataset, warranting further analysis.

## Data Availability

The datasets presented in this study can be found in online repositories. The names of the repository/repositories and accession number(s) can be found below: Mistletoe chronobiology: 27 years of daily metabolomic fingerprinting (dataset 1) DOI: 10.5281/zenodo.11032266.

## References

[B1] Ambrosio R. L. Gratino L. Mirino S. Cocca E. Pollio A. Anastasio A. (2020). The bactericidal activity of protein extracts from loranthus europaeus berries: a natural resource of bioactive compounds. Antibiotics 9, 47–14. 10.3390/antibiotics9020047 32012849 PMC7168301

[B2] Ameer O. Z. Salman I. M. Quek K. J. Asmawi M. Z. (2015). Loranthus ferrugineus: a mistletoe from traditional uses to laboratory bench. J. Pharmacopuncture 18, 7–18. 10.3831/KPI.2015.18.001 PMC4379471 25830054

[B3] Aono Y. Kazui K. (2008). Phenological data series of cherry tree flowering in Kyoto, Japan, and its application to reconstruction of springtime temperatures since the 9th century. Int. J. Climatol. 28, 905–914. 10.1002/joc.1594

[B4] Arganda-Carreras I. Kaynig V. Rueden C. Eliceiri K. W. Schindelin J. Cardona A. (2017). Trainable Weka Segmentation: a machine learning tool for microscopy pixel classification. Bioinform 33, 2424–2426. 10.1093/bioinformatics/btx180 28369169

[B5] Asada T. Warner B. G. Banner A. (2003). Growth of mosses in relation to climate factors in a hypermaritime coastal peatland in British Columbia, Canada. J. Bryol. 106, 516–527. 10.1639/0007-2745(2003)106[516:gomirt]2.0.co;2

[B6] Athmann M. Bornhütter R. Busscher N. Doesburg P. Geier U. Mergardt G. (2021). An update on image forming methods: structure analysis and Gestalt evaluation of images from rocket lettuce with shading, N supply, organic or mineral fertilization, and biodynamic preparations. Agric 12, 307–323. 10.1007/s13165-021-00347-1

[B7] Barlow P. W. Fisahn J. Yazdanbakhsh N. Moraes T. A. Khabarova O. V. Gallep C. M. (2013). *Arabidopsis thaliana* root elongation growth is sensitive to lunisolar tidal acceleration and may also be weakly correlated with geomagnetic variations. Ann. Bot. 111, 859–872. 10.1093/aob/mct052 23532042 PMC3631336

[B8] Baumgartner S. (2016). “Mistelpräparate,” in Anthroposophische pharmazie. Editors Meyer U. Pa P. (Berlin: Salumed Verlag), 591–596.

[B9] Baumgartner S. Fluckiger H. Kunz M. Scherr C. Urech K. (2014). Evaluation of preclinical assays to investigate an anthroposophic pharmaceutical process applied to mistletoe (*Viscum album* L.) extracts. Evid. Based Complement. Altern. Med. 2014, 620974. 10.1155/2014/620974 PMC4024402 24876872

[B10] Buda A. Zawadzkia T. Krupaa M. Stolarza M. Okulskib W. (2003). Daily and infradian rhythms of circumnutation intensity in Helianthus annuus. Physiol. Plant. 119, 582–589. 10.1046/j.1399-3054.2003.00198.x

[B11] Busscher N. Kahl J. Andersen J.-O. Huber M. Mergardt G. Doesburg P. (2010). Standardization of the biocrystallization method for carrot samples. Biol. Agric. Hortic. 27, 1–23. 10.1080/01448765.2010.10510427

[B12] Büssing A. (2000). “Biological and pharmacological properties of Viscum album L,” in Mistletoe: the genus Viscum. Editor Büssing A. (Amsterdam, The Netherlands: Harwood academic publishers), 123–182.

[B13] Cabrera J. (2006) Texture analyzer. Available at: http://rsb.info.nih.gov/ij/plugins/texture.html (Accessed July 07, 2006).

[B14] Christensen B. T. Thomsen I. K. Eriksen J. (2022). The Askov long‐term field experiment (1894–2021) represents a unique research platform^#^ . J. Plant Nutr. Soil Sci. 185, 187–201. 10.1002/jpln.202100354

[B15] Derbidge R. Baumgartner S. Heusser P. (2016). Mistletoe berry outline mapping with a path curve function and recording the circadian rhythm of their phenotypic shape change. Front. Plant Sci. 7, 1749. 10.3389/fpls.2016.01749 27933073 PMC5122707

[B16] Derbidge R. Feiten L. Conradt O. Heusser P. Baumgartner S. (2013). Assessment of shape changes of mistletoe berries: a new software approach to automatize the parameterization of path curve shaped contours. PLoS One 8, e60522. 10.1371/journal.pone.0060522 23565255 PMC3614953

[B17] Doesburg P. Andersen J. O. Scherr C. Baumgartner S. (2019). Empirical investigation of preparations produced according to the European Pharmacopoeia monograph 1038. Eur. J. Pharm. Sci. 137, 104987. 10.1016/j.ejps.2019.104987 31295547

[B18] Dorka R. Miersch O. Wasternack C. Weik P. (2007). Chronobiological phenomena and seasonal changes in jasmonate levels during the course of the year and under constant conditions in mistletoe (*Viscum album* L.). Phytomedicine 14, 15. 10.1016/j.phymed.2007.07.014 17140783

[B19] Flückiger H. Baumgartner S. (2002). Formveränderungen reifender mistelbeeren. Elem. D. N. 77, 2–15. 10.18756/edn.79.2

[B20] Fyfe A. (1969). The mistletoe in the cycle of the seasons. Br. Hom J. 58, 227–240. 10.1016/s0007-0785(69)80068-2

[B21] Fyfe A. (1975). Moon and plant - capillary dynamic studies. Arlesheim Soc. Cancer Res.

[B22] Gerdol R. (1996). The seasonal growth pattern of Sphagnum magellanicum Brid. in different microhabitats on a mire in the southern Alps (Italy). Oecol. Mont. 5, 13–20.

[B23] Gupta G. Kazmi I. Afzal M. Rahman M. Saleem S. Ashraf M. S. (2012). Sedative, antiepileptic and antipsychotic effects of *Viscum album* L. (Loranthaceae) in mice and rats. J. Ethnopharmacol. 141, 810–816. 10.1016/j.jep.2012.03.013 22449438

[B24] Haralick R. M. Shanmugam K. Dinstein I. (1973). Textural features for image classification. IEEE Trans. Syst. Man. Cybern. SMC-3, 610–621. 10.1109/tsmc.1973.4309314

[B25] Hemming D. L. Abernethy R. Armitage C. Bolmgren K. Myneni R. Park T. (2018). Sidebar 2.3. Phenol. Terr. Freshw. Prim. Prod. BAMS 99, 63–S66.

[B26] Inhetveen H. Schmitt M. Spieker I. (2021) Passion und Profession; Pionierinnen des ökologischen Landbaus. München: Oekom Verlag.

[B27] Kienle G. Kiene H. (2007). Complementary cancer therapy: a systematic review of prospective clinical trials on anthroposophic mistletoe extracts. Eur. J. Med. Res. 12, 103–119. 17507307

[B28] Kienle G. G. Kiene H. (2003) Die Mistel in der Onkologie. Fakten und konzeptionelle Grundlagen. Stuttgart: Schattauer.

[B29] Kienle G. S. Grugel R. Kiene H. (2011). Safety of higher dosages of *Viscum album* L. in animals and humans--systematic review of immune changes and safety parameters. BMC Complement. Altern. Med. 11, 72. 10.1186/1472-6882-11-72 21871125 PMC3180269

[B30] Kokornaczyk M. O. Bodrova N. B. Baumgartner S. (2021). Diagnostic tests based on pattern formation in drying body fluids - a mapping review. Colloids Surf. B. Biointerfaces 208, 112092. 10.1016/j.colsurfb.2021.112092 34537495

[B31] Kokornaczyk M. O. Primavera F. Luneia R. Baumgartner S. Betti L. (2016). Analysis of soils by means of Pfeiffer’s circular chromatography test and comparison to chemical analysis results. Biol. Agric. Hortic. 33, 143–157. 10.1080/01448765.2016.1214889

[B32] Kolisko E. Kolisko L. (1978) Agricolture of tomorrow. Rudge cottage, edge, stroud, glos. Gloucester: Kolisko Archive.

[B33] Kolisko L. (1953). Die kapillar-dynamolysis. Hippokrates 24, 130–135. 13068841

[B34] Koopmans A. (1972). Jahreszeitliche Veränderungen im Kristallisationsbild von Viscum Mali. Elem. D. N. 16, 43–52. 10.18756/edn.16.43

[B35] Krause H.-M. Stehle B. Mayer J. Mayer M. Steffens M. Mäder P. (2022). Biological soil quality and soil organic carbon change in biodynamic, organic, and conventional farming systems after 42 years. ASD 42, 117. 10.1007/s13593-022-00843-y

[B36] Larsen H. S. Laursen J. Pind N. Pyskow B. (2013). Development of a comprehensive method for steigbild characterization, analysis and interpretation. Elem. D. N. 99, 51–75. 10.18756/edn.99.51

[B37] Lindholm T. (1990). Growth dynamics of the peat moss Sphagnum fuscum on hummocks on a raised bog in southern Finland. Ann. Bot. Fenn. 27, 67–78.

[B38] Loef M. Walach H. (2020). Quality of life in cancer patients treated with mistletoe: a systematic review and meta-analysis. BMC Complement. Med. Ther. 20, 227. 10.1186/s12906-020-03013-3 32690087 PMC7370416

[B39] Mader P. Fliessbach A. Dubois D. Gunst L. Fried P. Niggli U. (2002). Soil fertility and biodiversity in organic farming. Science 296, 1694–1697. 10.1126/science.1071148 12040197

[B40] Maldacker J. (2006). Preclinical investigations with mistletoe (*Viscum album* L.) extract Iscador. Arzneim.-Forsch. 56, 497–507. 10.1055/s-0031-1296817 16927531

[B41] Mancuso S. Shabala S. (2006) Rhythms in plants. Dynamic responses in a dynamic environment. Berlin Heidelberg: Springer-Verlag.

[B42] Mcclung C. R. (2006). Plant circadian rhythms. Plant Cell 18, 792–803. 10.1105/tpc.106.040980 16595397 PMC1425852

[B43] Melzer J. Iten F. Hostanska K. Saller R. (2009). Efficacy and safety of mistletoe preparations (*Viscum album*) for patients with cancer diseases. A systematic review. Forsch. Komplementarmed. 16, 217–226. 10.1159/000226249 19729932

[B44] Mironov V. L. Kondratev A. Y. (2017). Peat moss Sphagnum riparium follows a circatrigintan growth rhythm *in situ*: a case report. Chronobiol. Int. 34, 981–984. 10.1080/07420528.2017.1329208 28574290

[B45] Mironov V. L. Kondratev A. Y. Mironova A. V. (2020). Growth of Sphagnum is strongly rhythmic: contribution of the seasonal, circalunar and third components. Physiol. Plant. 168, 765–776. 10.1111/ppl.13037 31613995

[B46] Moghadamtousi S. Z. Hajrezaei M. Abdul Kadir H. Zandi K. (2013). Loranthus micranthus linn.: biological activities and phytochemistry. Evid. Based Complement. Altern. Med. 2013, 273712. 10.1155/2013/273712 PMC3784240 24109490

[B47] Nievola C. C. Carvalho C. P. Carvalho V. Rodrigues E. (2017). Rapid responses of plants to temperature changes. Temperature 4, 371–405. 10.1080/23328940.2017.1377812 PMC5800372 29435478

[B48] O’byrne M. Ghosh B. Pakrashi V. Schoefs F. (2012). Texture analysis based detection and classification of surface features on ageing infrastructure elements. BCRI2012 Bridge & Concr. Res. Cork, Irel.

[B49] Ogal H. P. (2005). Schmerztherapie von Tumorerkrankungen. Schweiz. Z. Fur Ganzheitsmed. 7-8, 401–407.

[B50] Omeje E. O. Khan M. P. Osadebe P. O. Tewari D. Khan M. F. Dev K. (2014). Analysis of constituents of the eastern Nigeria mistletoe, Loranthus micranthus linn revealed presence of new classes of osteogenic compounds. J. Ethnopharmacol. 151, 643–651. 10.1016/j.jep.2013.11.029 24269773

[B51] Ostermann T. Appelbaum S. Poier D. Boehm K. Raak C. Bussing A. (2020). A systematic review and meta-analysis on the survival of cancer patients treated with a fermented *Viscum album* L. Extract (iscador): an update of findings. Complement. Med. Res. 27, 260–271. 10.1159/000505202 31927541

[B52] Ostermann T. Raak C. Bussing A. (2009). Survival of cancer patients treated with mistletoe extract (Iscador): a systematic literature review. BMC Cancer 9, 451. 10.1186/1471-2407-9-451 20021637 PMC2804713

[B53] Pfeiffer E. (1984) Chromatography applied to quality testing. Wyoming, RI: bio-Dynamic Literature.

[B54] Ramm H. Urech K. Scheibler M. Grazi G. (2000). “Cultivation and development of *Viscum album* L,” in MISTLETOE the genus Viscum (Amsterdam: Harwood academic publishers), 75–94.

[B55] Scheer R. Scheffler A. Errenst M. (1992). Two harvesting times, summer and winter: are they essential for preparing pharmaceutics from mistletoe (*Viscum album*)? Planta Med. 58, 594–599. 10.1055/s-2006-961584

[B56] Schindelin J. Arganda-Carreras I. Frise E. Kaynig V. Longair M. Pietzsch T. (2012). Fiji: an open-source platform for biological-image analysis. Nat. Methods 9, 676–682. 10.1038/nmeth.2019 22743772 PMC3855844

[B57] Sharquie K. E. Noaimi A. A. Saleh B. A. (2016). <i&amp;gt;Loranthus europaeus&amp;lt;/i&amp;gt; as an Alternative Medicine in Treatment of Acute Cutaneous Lesihmaniasis: review Article. J. Cosmet. Dermatological Sci. Appl. 06, 24–33. 10.4236/jcdsa.2016.61004

[B58] Singh B. N. Saha C. Galun D. Upreti D. K. Bayry J. Kaveri S. V. (2016). European *Viscum album*: a potent phytotherapeutic agent with multifarious phytochemicals, pharmacological properties and clinical evidence. RSC Adv. 6, 23837–23857. 10.1039/c5ra27381a

[B59] Sivasankar J. Thimmaiah A. (2021). Lunar rhythms in agriculture - review on scientific perspectives. Int. J. Complement. Altern. Med. 14, 81–85. 10.15406/ijcam.2021.14.00536

[B60] Skjerbaek K. Zalecka A. Kahl J. Huber M. Doesburg P. (2004) Triangle report Nr. 2: development and characterization of the capillary dynamolysis method for food quality analysis. Galten (DK): biodynamic research association Denmark, BRAD (Denmark) University of Kassel, Kassel (Germany), Louis Bolk Instituut (LBI) (Netherlands).

[B61] Steffen W. (1983). Untersuchungen zu den experimentellen und physikalisch-chemischen Grundlagen der Steigbildmethode. Elem. D. N. 38, 36–49. 10.18756/edn.38.36

[B62] Steiner R. (1961). “Geisteswissenschaft und Medizin, 13th lecture, Dornach, 2. April 1920,” in Geisteswissenschaft und Medizin. Editor Zbinden H. W. 4th ed. (Dornach, Switzerland: Rudolf Steiner Nachlassverwaltung), 210–227.

[B63] Steiner R. Wegman I. (1925) Grundlegendes für eine Erweiterung der Heilkunst nach geisteswissenschaftlichen Erkenntnissen. Dornach, Switzerland: Rudolf Steiner Nachlassverwaltung.

[B64] Suveren E. Baxter G. F. Iskit A. B. Turker A. U. (2017). Cardioprotective effects of *Viscum album* L. subsp. album (European misletoe) leaf extracts in myocardial ischemia and reperfusion. J. Ethnopharmacol. 209, 203–209. 10.1016/j.jep.2017.07.010 28689799

[B65] Szurpnicka A. Kowalczuk A. Szterk A. (2020). Biological activity of mistletoe: *in vitro* and *in vivo* studies and mechanisms of action. Arch. Pharm. Res. 43, 593–629. 10.1007/s12272-020-01247-w 32621089 PMC7340679

[B66] Unluturk M. S. Kucukyasar S. Pazir F. (2021). Classification of organic and conventional olives using convolutional neural networks. Neural. comput. Appl. 33, 16733–16744. 10.1007/s00521-021-06269-z

[B67] Unluturk S. M. Unluturk S. Pazir F. Abdollahi F. (2011). Capillary dynamolysis image discrimination using neural networks. Int. J. Inf. Tech. Softw. Eng. 01. 10.4172/2165-7866.1000101

[B68] Upadhyay H. Juneja A. Turabieh H. Malik S. Gupta A. Bitsue Z. K. (2022). Exploration of crucial factors involved in plants development using the fuzzy AHP method. Math. Probl. Eng. 2022, 1–9. 10.1155/2022/4279694

[B69] Urech K. Baumgartner S. (2015). Chemical constituents of *Viscum album* L.: implications for the pharmaceutical preparation of mistletoe. Transl. Res. Biomed. 4, 11–23. 10.1159/000375422

[B70] Urech K. Jäggy C. Schaller G. (2009). “Räumliche und zeitliche Dynamik der Viscotoxin-und Mistellektingehalte in der Mistel,” in Die Mistel in der Tumortherapie 2 - Aktueller Stand der Forschung und klinische Anwendung. Editors Scheer R. Alban S. Becker H. Holzgrabe U. Kemper F. Kreis W. (Essen: KVC Verlag), 67–78.

[B71] Venkat A. Muneer S. (2022). Role of circadian rhythms in major plant metabolic and signaling pathways. Front. Plant Sci. 13, 836244. 10.3389/fpls.2022.836244 35463437 PMC9019581

[B72] Yazaki T. Yabe K. (2012). Effects of snow-load and shading by vascular plants on the vertical growth of hummocks formed by Sphagnum papillosum in a mire of Northern Japan. Plant Ecol. 213, 1055–1067. 10.1007/s11258-012-0065-x

[B73] Zalecka A. Kahl J. Doesburg P. Pyskow B. Huber M. Skjerbaek K. (2010). Standardization of the steigbild method. Biol. Agric. Hortic. 27, 41–57. 10.1080/01448765.2010.10510429

[B74] Zuber D. (2004). Biological flora of central europe: *Viscum album* L. Flora 199, 181–203. 10.1078/0367-2530-00147

