# Supplementary material for: Chronobiology of Viscum album L.: a time series of daily metabolomic fingerprints spanning 27 years
Source: Front Physiol. 2024 May 27;15:1396212. doi: 10.3389/fphys.2024.1396212 (PMC11163206; doi:10.3389/fphys.2024.1396212)
Supplement: Supplementary file 1 [file Table1.docx]

Supplementary Material

**Table S1: List of the selected weather variables provided by Meteo Swiss.**

|  |  |  |
| --- | --- | --- |
| Parameter | Description | Unit |
| fkl010d0 | Wind speed scalar; daily mean | m/s |
| fu2010d0 | Wind speed scalar; daily mean | kt |
| fu3010d0 | Wind speed scalar; daily mean | km/h |
| hns000d0 | Fresh snow; daily total 6 UTC - 6 UTC following day | cm |
| hns002d0 | Fresh snow; 2-day-total - 48 h | cm |
| hns003d0 | Fresh snow; 3-day-total - 72 h | cm |
| hns004d0 | Fresh snow; 4-day-total - 96 h | cm |
| hns005d0 | Fresh snow; 5-day-total - 120 h | cm |
| hns010d0 | Fresh snow; 10-day-total - 240 h | cm |
| hto000d0 | Snow depth; morning measurement at 6 UTC | cm |
| nto008d0 | Dark day; Mean cloud cover over 80 % | y/n |
| prestad0 | Atmospheric pressure at barometric altitude (QFE); daily mean | hPa |
| prestadn | Atmospheric pressure at barometric altitude (QFE); daily minimum | hPa |
| prestadx | Atmospheric pressure at barometric altitude (QFE); daily maximum | hPa |
| pva200d0 | Vapour pressure 2 m above ground; daily mean | hPa |
| rhs150d0 | Precipitation; homogeneous daily total (conventional) 0540 - 0540 following day (statistically derived) | mm |
| rre002d0 | Precipitation; 2-day-total, 6 UTC following day - 48 h | mm |
| rre003d0 | Precipitation; 3-day-total, 6 UTC following day - 72 h | mm |
| rre004d0 | Precipitation; 4-day-total, 6 UTC following day - 96 h | mm |
| rre005d0 | Precipitation; 5-day-total, 6 UTC following day - 120 h | mm |
| rre010d0 | Precipitation; 10-day-total, 6 UTC following day - 240 h | mm |
| rre150n0 | Precipitation; semi-daily total 18 UTC - 6 UTC | mm |
| sre000d0 | Sunshine duration; daily total | min |
| sremaxdv | Sunshine duration; in relation to absolute daily total | % |
| su2000d0 | Sunshine duration; daily total | h |
| ths200d0 | Air temperature 2 m above ground; homogeneous daily mean (statistically derived) | °C |
| ths200dn | Air temperature 2 m above ground; homogeneous daily minimum (statistically derived) | °C |
| ths200dv | Air temperature 2 m above ground; deviation of the homogeneous daily mean (statistically derived) to the norm 6190 | °C |
| ths200dx | Air temperature 2 m above ground; homogeneous daily maximum (statistically derived) | °C |
| ths2dndv | Air temperature 2 m above ground; deviation of the homogeneous daily minimum (statistically derived) to the norm 6190 | °C |
| ths2dxdv | Air temperature 2 m above ground; deviation of the homogeneous daily maximum (statistically derived) to the norm 6190 | °C |
| tre200d0 | Air temperature 2 m above ground; daily mean | °C |
| tre200dn | Air temperature 2 m above ground; daily minimum | °C |
| tre200dx | Air temperature 2 m above ground; daily maximum | °C |
| ure200d0 | Relative air humidity; 2 m above ground; daily mean | % |
| w2p001d0 | Day with rain and snow | y/n |
| w3p001d0 | Day with graupel | y/n |
| w4pclod0 | Day with near thunderstorm | y/n |
